# Status of the Multidrug Resistance-1 Gene of *Plasmodium falciparum* in Four Malaria Epidemiological Strata, Two Decades after the Abolition of Chloroquine as First-Line Treatment for Uncomplicated Malaria in Cameroon

**DOI:** 10.1155/2023/6688380

**Published:** 2023-07-01

**Authors:** David Denis Sofeu-Feugaing, Fabrice Nkengeh Ajonglefac, Marcel Nyuylam Moyeh, Tobias Obejum Apinjoh, Marianne Elodie Essende, Gilchrist Destin Talla Kouam, Stephen Mbigha Ghogomu

**Affiliations:** ^1^Department of Biochemistry and Molecular Biology, University of Buea, Buea, Cameroon; ^2^Department of Chemical and Biological Engineering, School of Engineering, University of Bamenda, Bamenda, Cameroon

## Abstract

Drug-resistant malaria parasites pose a threat to global malaria control efforts, and it is important to know the extent of these drug-resistant mutations in each region to determine appropriate control measures. Chloroquine (CQ) was widely used in Cameroon for decades, but its declining clinical efficacy due to resistance prompted health authorities in 2004 to resort to artemisinin-based combination therapy (ACT) as the first-line treatment for uncomplicated malaria. Despite numerous efforts to control malaria, it persists, and the emergence and spread of resistance to ACTs make the development of new drugs or the possible reintroduction of discontinued drugs increasingly urgent. Malaria-positive blood samples were collected from 798 patients on Whatman filter paper to determine the status of resistance to CQ. DNA was extracted by boiling in Chelex and analysis of *Plasmodium* species. Four hundred *P. falciparum* monoinfected samples, 100 per study area, were amplified by nested PCR, and allele-specific restriction analysis of Pfmdr1 gene molecular markers was performed. Fragments were analyzed using a 3% ethidium bromide-stained agarose gel. *P. falciparum* was the most abundant *Plasmodium* species, accounting for 87.21% of *P. falciparum* monoinfections only. No infection with *P. vivax* was detected. The majority of samples contained the wild type for all 3 SNPs evaluated on the Pfmdr1 gene with N86, Y184, and D1246 accounting for 45.50%, 40.00%, and 70.00%, respectively. The most abundant haplotype observed was the Y184D1246 double wild type at 43.70%. The results suggest that *P. falciparum* is the major infecting species and that *P. falciparum* species with the susceptible genotype are gradually recapturing the parasite population.

## 1. Introduction

Malaria remains a widespread public health threat in sub-Saharan Africa, which accounts for 95% of global malaria morbidity and mortality [1]. Infants, children under 5, pregnant women, HIV-positive patients, and unimmunized migrants from nonmalarious communities are most affected [2, 3]. In 2022, there were 247 million estimated malaria cases and 619,000 malaria-related deaths worldwide [4]. In Cameroon, there were almost 6.67 million cases and over 14,000 deaths [4]. Unfortunately, there is no malaria-free zone in Cameroon, and almost 20 million people live in areas with high malaria transmission, 41% of whom are expected to have at least one episode per year [5]. Chemotherapy is currently one of the mainstays of malaria control. However, the constant emergence of resistant strains of malaria parasites poses a threat to global malaria control efforts. Chloroquine (CQ) was the drug of choice for the treatment of uncomplicated malaria in Cameroon until 2002 when the national malaria control banned the importation and use of the drug [6]. It accumulates in the parasite's food vacuole and causes the accumulation of heme, a toxic by-product of haemoglobin breakdown, and subsequent death of the parasite [7–9]. The characteristic of resistance is a decrease in the amount of chloroquine that accumulates in the food vacuole, as a consequence of mutations in two important transport protein genes: chloroquine resistance transporter (CRT), the main transporter of CQ, and multidrug resistance protein 1 (MDR1), which plays a modulatory role in CQ transport, but an important role in transporting other aminoquinolines such as AQ [10, 11]. Although no clear consensus has been reached on the mechanism of chloroquine resistance, there is ample evidence of a positive correlation with mutations in the chloroquine resistance transporter gene (Pfcrt). This gene encodes a vacuolar transmembrane protein responsible for transporting the drug into the parasite's food vacuole [12, 13]. All chloroquine-resistant parasite lines, as well as resistant clinical isolates, carry lysine to threonine mutation at codon 76 (K76T mutation), and this has therefore been used as a surrogate marker to monitor chloroquine resistance in field studies [14]. Aminoquinoline drug sensitivity is further modulated by mutations in *P. falciparum* multidrug resistance gene 1 (Pfmdr1). The SNPs involved include N86Y, Y184F, S1034C, N1042D, and D1246Y [15, 16]. *Plasmodium* resistance to CQ was first reported in Africa in the 1970s [17, 18] and 1985 in Cameroon [19]; a series of clinical trials revealed a general ineffectiveness of CQ throughout the country, leading to the gradual withdrawal of CQ and the use of amodiaquine (AQ) from 2002 to 2004, which was quickly replaced by artesunate-amodiaquine, with artemether-lumefantrine as an alternative ACT since 2006 [20, 21]. The introduction of ACTs drastically reduced worldwide malaria deaths by over 35% (approximately 329,000 deaths) from 2006 to 2019 [22, 23]. Even if we disregard the 10% increase in malaria-related deaths since 2019 [4] which has been attributed to the COVID-19 pandemic, recent success in global malaria control is threatened by the emergence of resistance to ACTs and fears of further spread of the disease in vulnerable areas, particularly in sub-Saharan Africa, where malaria transmission rates are very high. In addition, malaria morbidity and mortality in Cameroon have declined steadily since 2006 but have experienced a sudden increase in the last three years, albeit by less than 7% [24]. This necessitates the development of new therapeutics or the possible reintroduction of old drugs, ideally in combination therapy [1, 25]. The re-emergence of the wild type N86, Y184, and D1246 alleles of the Pfmdr1 gene and strains susceptible to CQ has been documented in some localities across the continent [26, 27]. Several studies have shown a decline in the mutant population. A nationwide study representing the different ecological and transmission settings of Cameroon has not been performed. Resistance of *P. falciparum* to CQ has long denied healthcare end users' access to cheap and effective antimalarials.

In this study, we attempt to determine the status of aminoquinoline resistance in several key Cameroonian localities, including the western plateau strata (Bafoussam), the southern equatorial strata (Bafia), and the coastal strata (Mount Cameroon Area (MCA) and Douala) adjacent to the two aforementioned strata to the north and east, respectively. Known as malaria epidemiological strata, these geographically distinct ecological zones within the national territory tend to have different malaria vector, parasitology, and transmission patterns, as previously described [28, 29]. The three carboxyl terminal mutations (S1034C, N1042D, and D1246Y) are more common in South American isolates than the amino terminal mutations (N86Y and Y184F), which are more common in Asian and African parasites. However, D1246Y is present in approximately 3% of the 1,502 African genomes recently sequenced by MalariaGEN [30]. Although the Cameroonian government banned the import and use of CQ in 2002, thereby removing the pressure on the population from CQ drugs, it still adopted AQ in combination with artesunate as well as artemether-lumefantrine (AL) for the treatment of uncomplicated falciparum malaria in Cameroon. The presence of AQ, another 4-aminoquinoline, equally maintained the drug pressure. Despite increasing resistance to CQ, the *in vivo* efficacy of AQ combination therapy in Cameroon has shown to be good [31]. Across Africa, it has been established that the K76T mutation in the Pfcrt gene is present in all CQ-resistant strains and that mutations in the Pfmdr1 gene contribute to the modulation of this resistance [14]. Mutations in the Pfmdr1 gene appear to be an essential component of resistance to the structurally related drug quinine. In Cameroon, quinine is still maintained as the drug of choice for the treatment of severe falciparum malaria. A strong association between possession of the wild-type form of Pfmdr1, amplification of Pfmdr1, and resistance to hydrophobic drugs, such as arylamino alcohol mefloquine and endoperoxide artemisinin derivatives in field isolates, has been observed [15, 16]. Using ACTs selected for the wild-type Pfmdr1 genotype, monitoring changes in the prevalence of Pfmdr1 SNPs could provide an early warning of the emergence of resistance to ACT. Therefore, the aim of the current investigation is to understand the impact of stopping CQ 20 years ago and using ASAQ as first-line therapy on recovery of CQ-sensitive Pfmdr1 gene variations. We therefore worked on the hypothesis that *P. falciparum* species with CQ-sensitive variants of Pfmdr1 recolonized the parasite population in Cameroon. Based on previously established molecular markers of *P. falciparum* resistant to CQ and related aminoquinolines, we determined the prevalence of 86Y, 184F, and 1246Y mutants and the corresponding wild-type markers (N86, Y184, and D1246) of the Pfmdr1 gene. Finally, haplotype construction was performed to determine the most prevalent combination of markers in the population.

## 2. Methodology

This study followed a logical sequence of methodology: patient recruitment and sample collection, characterization of *Plasmodium* species, and PCR and allele-specific restriction analysis of molecular markers on the Pfmdr1 gene of *P. falciparum*, followed by data analysis and haplotype construction.

### 2.1. Patient Recruitment and Sample Collection

#### 2.1.1. Study Area

**Figure 1:**
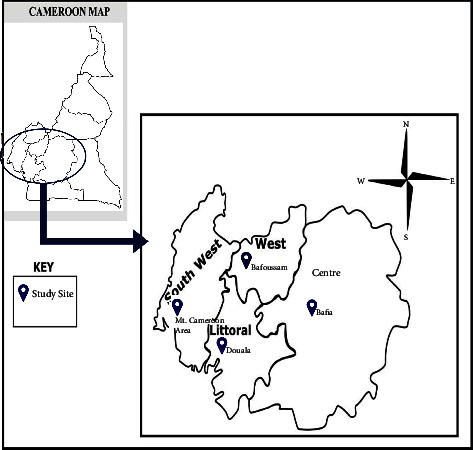
Map showing the location of all four study sites.

This study was conducted at Douala (4.050°N and 9.683°E) in the equatorial forest zone, Bafoussam (5.4808°N and 10.4284°E) in the Guinea savanna zone, Bafia (4.450°N and 11.140°E) in the forest-savanna transition zone, and the Mount Cameroon Area (MCA), defined as Mutengene, Limbe, and Buea (4,010°N and 9,140°E) in the tropical rainforest zone [32, 33] (Figure 1). All have high and perennial malaria transmission [34, 35].

#### 2.1.2. Ethical Consideration

The ethical authorisation was obtained from the Ethical Review Board of the Faculty of Health Sciences, University of Buea, with the following trial registration number: Ref N°: 2019/1018-08/UB/SG/IRB/FHS (supplementary material S1), and administrative approval was obtained from the Ministry of Public Health through the various Regional Delegations of Public Health (R11/MINSANTE/SWR/RDPH/PS/268/784). In addition, permission was obtained from the director of various hospitals where samples were collected, and participants or guardians signed an informed consent form before sample collection.

#### 2.1.3. Sample Collection

Patients presenting at the outpatient department with signs and symptoms suggestive of malaria, at “Hôpital Regional” and “Hôpital de District de la Mifi, Famla” in Bafoussam, “Hôpital de District de Bonasssama” and “Hôpital Laquintinie d'Akwa” in Douala, “Hôpital de District de Bafia” and nonsecour private health centre in Bafia, Buea Regional Hospital, Baptist Hospital Mutengene, and Limbe Regional Hospital at the MCA, had their informed consent obtained, after which demographic and basic clinical data were recorded on individual clinical case report forms. Children less than 5 month old, pregnant women, and individuals with signs and symptoms suggestive of severe malaria were excluded from the study. Blood smears and dried blood spots (DBS) were prepared from capillary blood obtained by fingerprick on sterile, glass slides and labelled filter paper (Whatman® No.3, Sigma-Aldrich, Germany) and air drying. Potential participants were screened for malaria by light microscopy using Giemsa staining. For each blood smear preparation, parasite density was determined by counting parasites against at least 200 white blood cells (WBCs) and assuming an average of 8000 WBCs/L. Each DBS was stored separately in labelled opaque envelopes alongside desiccant and kept away from light and humidity and transported to the molecular and cell biology laboratory of the Biotechnology Unit of the University of Buea, where parasite DNA was extracted from the DBS using the Chelex®-100 (Bio-Rad, Berkeley California, USA) saponin method, as previously described [36]. The extracted DNA was stored at −20°C till use.

### 2.2. Characterization of *P. falciparum* Species

Species-specific primers (Inqaba Biotec, Pretoria, South Africa) targeting the small subunit 18S rRNA *Plasmodium* gene were used to amplify and characterize *Plasmodium* species by nested PCR, as previously described [37]. The resulting amplicons were separated on a 2% agarose gel stained with ethidium bromide and visualised using a gel documentation system (Molecular Imager® Gel DocTM XR + System with Image LabTM Software, Bio-Rad, Berkeley, California, USA). PCR was repeated for all negative samples. Samples that were negative after two PCRs, samples with mixed infections of *P. falciparum* and other species, and monoinfections with species other than *P. falciparum* were removed from the study. Only samples with *P. falciparum* monoinfection were used for subsequent experiments.

### 2.3. Nested PCR and Allele-Specific Restriction Analysis of *P. falciparum* Monoinfected Samples

Portions of the Pfmdr1 gene comprising codons 86, 184, and 1246 were amplified by nested PCR as previously described [38], using sequence-specific primers purchased from Inqaba Biotech (Pretoria, South Africa) for 400 randomly selected samples, 100 per study site; randomisation was performed with the aid of a computer-generated list. The amplicons were analyzed by restriction fragment length polymorphism (RFLP) using the restriction endonucleases ApoI, DraI, and BglII, as previously described [38]. The restrictive fragments were resolved on a 3% agarose gel stained with ethidium bromide and later visualised in a gel documentation system (Molecular Imager® Gel DocTM XR + System with Image LabTM Software, Bio-Rad, Berkeley, California, USA).

### 2.4. Data Analysis and Haplotype Construction

The baseline demographic and clinical data of study participants were analyzed using descriptive statistics, whereas the Pearson correlation coefficient was used to evaluate the relationship between clinical and demographic parameters. The prevalence of each marker polymorphism and haplotype was compared using the chi-square test. Statistical significance was assumed at *p* < 0.05. All analysis was performed using the *R* statistical package. Finally, to ease data analysis during the analysis of SNPs, mixed infections with both a sensitive strain and a resistant strain were considered resistant, and in haplotype analysis, mixed infections were discarded.

## 3. Results

As shown in Table 1, a total of 798 microscopically positive malaria samples were collected (Douala = 209, Bafoussam = 200, Bafia = 174, and MCA = 215). The mean age of participants was 24 ± 18 years and ranged from 1 to 80 years. A total of 58.27% (465/798) participants were female. The geometric mean parasite density (GMPD) was 3347 asexual parasites/*μ*l.

PCR determination of species diversity was performed on all 798 microscopically positive samples. *P. falciparum* was most common at 96.62% (771/798) and was either monoinfection or mixed infection with other species. Monoinfection with *P. falciparum* alone accounted for 87.22% (696/798). *P. malariae* monoinfection was 2.88% (23/798). There was no monoinfection with *P. vivax* and *P. ovale* species; however, there was a 5.64% (45/798) mixed infection with *P. falciparum* and *P. malariae* and 3.26% (26/798) mixed infections with *P. falciparum* and *P. ovale*. A 0.50% (04/798) mixed infection with *P. malariae* and *P. ovale* was noted, while a 0.50% (04/798) triple infection with *P. falciparum*, *P. malariae*, and *P. ovale* was also observed (Table 2).

Allele-specific restriction analysis of the Pfmdr1 gene was performed only for samples monoinfected with *P. falciparum.* PCR amplification of all fragments was successful for 400 randomly selected samples, 100 per study site.  Table 3 shows the distribution of alleles obtained for the different drug-resistant markers on the Pfmdr1 gene in all study sites.

**Figure 2:**
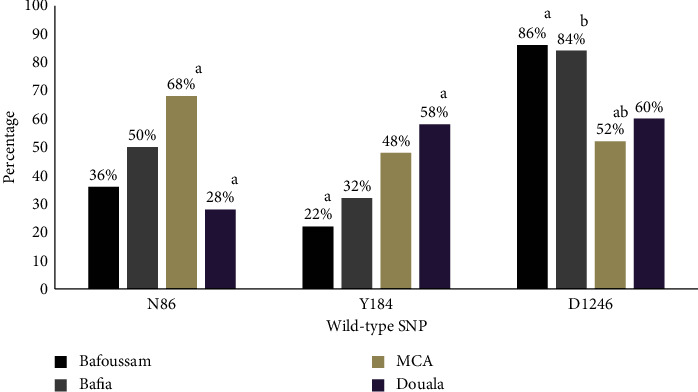
Distribution of SNPs in the Pfmdr1 gene per study site. At *P* < 0.05, the results of the chi-square test showed a significant difference in the prevalence of SNP variations in all areas. Furthermore, the main causes of these significant differences were identified by the post hoc analysis using Bonferroni correction to exclude type 1 errors. Similar superscript letters indicate values that are significantly different from each other when present. In addition, values with double superscript letters are significantly different from any value with at least one of the associated letters, but values with different or no superscript letters show no statistically significant difference. The modified *P* value = 0.00625 was used to compare post hoc *P* values.

In calculating the prevalence of SNPs, samples containing a mixture of CQ-sensitive and resistant variants of an SNP were considered mutants for that SNP. Therefore, the sensitive genotypes N86, Y184, and D1246 of the Pfmdr1 gene were detected in 45.50% (182/400), 40.00% (160/400), and 70.00% (280/400) of the field isolates, respectively (Table 3). The distribution of the various marker genotypes in the different study sites is presented in Figure 2.

**Figure 3:**
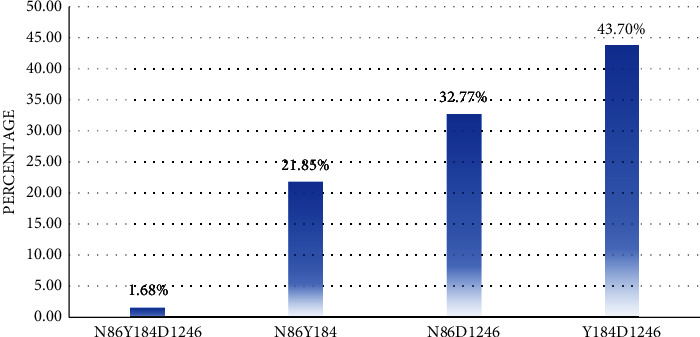
Prevalence of Pfmdr1 haplotypes in percentages in all study sites.

The haplotype analysis revealed double or triple mutant haplotypes as most of the mutants were observed as mixed infections and were not included in the haplotype analysis. This is also a very strong indicator of competition between wild and mutant parasite populations. NYD represents samples with only a triple wild type at codons 86, 184, and 1246; NY*∗* represents samples with a double wild type at codons 86 and 184; N*∗*D represents samples with a double wild type at codons 86 and 1246; and *∗*YD represents samples with a double wild type at codons 184 and 1246. The most abundant haplotype observed was the *∗*YD double wild type seen in 43.70% (104/240) of the samples closely followed by N*∗*D 32.77% (78/240) and NY*∗* 21.85% (52/240) double wild-type haplotypes; triple wild-type haplotype NYD was also observed in a very tiny fraction 1.68% (04/240) of samples (Figure 3). The distribution of the various haplotypes in the different study sites is summarized in Table 4.

## 4. Discussion

For decades before the emergence and spread of CQ-resistant parasite strains, CQ was a very cheap, safe, and effective drug for the treatment of uncomplicated falciparum malaria in Cameroon [20]. Mutations at positions N86Y, Y184F, S1034, N1042D, and D1246Y of *P. falciparum* multidrug resistance protein 1 (Pfmdr1) have been reported to modulate sensitivity and resistance to CQ and other aminoquinolones [16, 39–41]. The establishment of resistance to ACTs in Rwanda and fears of further spread in sub-Saharan Africa, where malaria transmission rates are very high, combined with the recent global increase of more than 4,000 deaths between 2018 and 2020, raise concerns and call for intervention. 4-aminoquinoline AQ is still used in Cameroon to treat uncomplicated malaria in combination with artesunate. Therefore, to extend the therapeutic life of this ACT, an in-depth analysis of the Pfmdr1 gene is needed.

In this study, we investigated the impact of the withdrawal of CQ from malaria treatment in Cameroon on molecular markers of aminoquinoline resistance on the Pfmdr1 gene and the different polymorphisms of this gene currently spreading in the population in four regions of Cameroon, covering three different Malaria epidemiological strata, to draw a clear current picture of the response of these marker genes in different ecological environments of Cameroon.

Molecular speciation of the study participants' samples by PCR revealed that *P. falciparum* was most prevalent either as a monoinfection or as a mixed infection with other species at 96.62%, which is in agreement with Kwenti et al. [42] and Russo et al. [43]. An 87.21% monoinfection with *P. falciparum* was also observed, which is consistent with 83.50% reported by [44, 45]. No cases of *P. vivax* were detected, which contradicts studies reporting a prevalence of *P. vivax* ranging from 4% to 14.9% in Cameroon [5, 46]. This may be because symptomatic patients were examined in this study, whereas both symptomatic and asymptomatic cases were examined in the other study. Coinfection of *P. falciparum* and *P. ovale* was found in 3.26% of cases, which is consistent with Payne et al. in 2007 [17]. Coinfection with *P. falciparum* and *P. malariae* was observed in 5.64% of samples. Coinfection with these *Plasmodium* species significantly alters the manifestation dynamics [47] which can lead to a nephrotic syndrome that is unresponsive to treatment and has a high mortality rate [48]. The nonnegligible incidence of *P. ovale* infections suggests that the country's current treatment policy, which primarily targets *P. falciparum* infections, should be revised to include primaquine treatment which will carter for hypnozoites resulting from *P. ovale* infection preceded by G6PD testing.

Chloroquine was removed from most stores in Cameroon after it was banned and replaced by ASAQ in 2002. Amodiaquine exerts the same drug pressure as chloroquine, as both are aminoquinolines. Among samples amplified by nested PCR followed by allele-specific restriction analysis without a prior study in Bafoussam and Bafia, the prevalence of Pfmdr1 mutants was 64.0%, and 50.0% for 86Y; 78.0%, and 68.0% for 184F; and 14.0%, and 16.0% for 1246Y. The results from Douala showed a large difference between 72.0%, 42.0%, and 40.0% in this study and 87.2%, 89.6%, and 100% in codons 86, 184, and 1246, respectively, as reported by Moyeh et al. [45]. This may be because their study combined samples from Buea and Douala, the discrepancies in sample sizes, and the fact that the samples for the current study were a hospital-based sample survey which may have affected the likelihood of encountering patients who were already unable to control their disease by self-medication, given the high prevalence of self-medication with antimalarials already reported in the region [49]. In the MCA, results showed a massive decrease in the prevalence of the mutation at codon 86 from 73.8% in 2010 [31] to 32.0%, from 72.0% in 2017 [50] to 52.0% at codon 184, except for an increase from 3.1% in 2018 [51] to 48.0% at codon 1246; although this discrepancy may be because our sample size included samples from three different locations (Limbe, Buea, and Mutengene) within the MCA, Moyeh et al. [51] only obtained samples from Mutengene. The most frequently observed haplotype was the ∗ YD double wild type, which occurred in 43.70% of samples, followed by N*∗*D 32.77% and NY*∗* 21.85% double wild-type haplotypes. Triple wild-type haplotype NYD was also observed in a very small proportion of 01.68% of the samples. The fact that the Pfmdr1 haplotypes observed were the triple and double wild-type NYD, *∗*YD, N*∗*D, and NY*∗*, and the fact that no mutant haplotypes were observed is an important indication that *P. falciparum* strains with the wild-type SNPs are dormant in the parasite population, suggesting a slow recovery to the CQ susceptible parasite strains. A trend of steady decrease was observed with the *∗*YD haplotype from the savanna (Bafoussam) at 68.18%, while in the forest-savanna transition zone (Bafia), it was 52.78%; in the forest-highland zone (MCA), it was 34.29%, and in the forest-lowland zone, it was 15.38%; this illuminates the reality that the different climatic conditions in these ecological environments influence the occurrence and spread of resistance [52–54], highlighting that drug resistance and ecological climate variability interact through the effects of warmer temperatures on transmission intensity, raising the possibility of synergistic interactions between warmer temperatures and drug resistance. The results generally suggest a slower recovery in susceptibility when compared with the chloroquine-resistant genotype in Malawi, which declined from 85% to 13% in 8 years between 1992 and 2000 [26]. Slower recovery could be considered an indicator of low susceptibility of *P. falciparum* isolates to amodiaquine [55], a compound of the current first-line treatment artesunate-amodiaquine.

## 5. Conclusion

The study revealed that *P. falciparum* parasites with the chloroquine-resistant genotype are gradually recolonizing the parasite population in our study sites but at a slower pace than reports from other countries within the sub-Saharan region have suggested. The much slower-paced recovery could be considered an indicator of the low susceptibility of *P. falciparum* isolates to amodiaquine, an important partner drug molecule of the current first-line treatment, artesunate-amodiaquine.

## 6. Limitations and Recommendations

This study was limited to four of the six malaria epidemiological regions of Cameroon, and further analysis of the current situation using data from other regions of the country may provide a better picture of the development dynamics and continued surveillance for markers of drug-resistant mutants of CQ in Cameroon. In addition, the government should consider a small switch from artesunate-amodiaquine to artemether-lumefantrine. This could recover a very important drug molecule that could be used as an emergency drug or as a partner drug in a new combination therapy in the event of a massive spread of ACT-resistant malaria in the country.

## Figures and Tables

**Table 1:** Demographics and baseline clinical data.

Items	Subitems	Results for each study site				
	Localities	Douala	MCA	Bafia	Bafoussam	Total
	*n* =	209	215	174	200	798
Gender	Male	88	94	63	88	333 (41.73%)
	Female	121	121	111	112	465 (58.27%)

Age (years)	Mean age ± std dev	25 ± 20	20 ± 15	31 ± 22	18 ± 18	24 ± 18
	Age range	1–80	2–65	1–80	1–78	1–80

Parasite density (asexual parasites/*μ*l)	Geo-mean parasitaemia	5031	2804	603	4951	3347
	Parasitaemia range	80–232000	91–90620	45–3433	160–80000	80–232000

Haemoglobin (Hgb) (g/dl)	Hgb conc. ± std dev	11.9 ± 2.2	12.4 ± 1.8	11.4 ± 2.6	12.3 ± 1.9	11.9 ± 22.0

**Table 2:** Distribution of parasite species among study sites.

	Douala	Bafoussam	Bafia	MCA	Total
*P. falciparum*	163/209	163/200	164/174	206/215	696
*P. malariae*	14/209	03/200	00/174	06/215	23
*P. falciparum* *+* *P. malariae*	20/209	17/200	05/174	03/215	45
*P. falciparum* *+* *P. ovale*	12/209	09/200	05/174	00/215	26
*P. malariae* *+* *P. ovale*	00/209	04/200	00/174	00/215	4
*P. falciparum* *+* *P. malariae* *+* *P. ovale*	00/209	04/200	00/174	00/215	4
Total	209	200	174	215	798

Proportions represent the number of field isolates with parasite species divided by the total number of samples analyzed for the species. When comparing the results between the four study sites, it was found that there was no significant difference between the proportions of falciparum parasites at the different study sites (*P* > 0.05).

**Table 3:** Distribution of SNPs in the Pfmdr1 gene in all study sites.

	Pfmdr1 (86)	Pfmdr1 (184)	Pfmdr1 (1246)
Wild type	45.50 (182/400)	40.00 (160/400)	70.00 (280/400)
Mutant	54.50 (218/400)	60.00 (240/400)	30.00 (120/400)

A sample is considered wild type for an SNP if it has the CQ-sensitive variant; mutants have the resistant variant and the mixed variants if they contain both. The numbers represent percentages, while the numbers in parentheses indicate the number of samples with each genotype in relation to the total number of samples successfully amplified.

**Table 4:** Distribution of haplotypes per study site.

	Haplotype analysis per study site			
	N86Y184	N86D1246	Y184D1246	N86Y184D1246
Douala	23.08 (12/52)	53.85 (28/52)	15.38 (12/52)	00.00 (00/52)
Bafoussam	00.00 (00/44)	31.82 (14/44)	68.18 (30/44)	00.00 (00/44)
Bafia	16.67 (12/72)	30.56 (22/72)	52.78 (38/72)	00.00 (00/72)
MCA	40.00 (28/70)	20.00 (14/70)	34.29 (24/70)	05.71 (04/70)
Total	21.85 (52/238)	32.77 (78/238)	43.70 (104/238)	01.68 (04/238)

Numbers represent percentages, while proportions in parentheses indicate the number of field isolates with haplotypes divided by the total number of samples analyzed.

## Data Availability

The publication contains all the information required to support the study's conclusions, and the authors are responsible for the outcomes reported. The references used for the data are appropriately credited.

## Supplementary Materials

Supplementary Materials S1: notice for ethical approval.
