# Supplementary material for: Status of the Multidrug Resistance-1 Gene of Plasmodium falciparum in Four Malaria Epidemiological Strata, Two Decades after the Abolition of Chloroquine as First-Line Treatment for Uncomplicated Malaria in Cameroon
Source: J Trop Med. 2023 Jul 1;2023:6688380. doi: 10.1155/2023/6688380 (PMC10329556; doi:10.1155/2023/6688380)
Supplement: Supplementary Materials — S1: notice for ethical approval. [file 6688380.f1.pdf]

FACULTY OF HEALTH SCIENCES- INSTITUTIONAL REVIEW BOARD

IRB00008917-US Office for Human Research Protection (OHRP)IORG007426

Chair : **Professor Choukem Simeon Pierre**

Secretary : **Associate Professor. Halle-Ekane Edie Gregory**

Your Ref ______________________

Our Ref: **2019/ 1018-08 /UB/SG/IRB/FHS**

Date: 6th November 2019

Notice of Ethical Approval

Application number: **1018-08**

Principal Investigator: **AJONGLEFAC NKENGEH FABRICE**

Study Title: **Assessing the prevalence of *pfmdr1* Y86, F184 and Y1246 molecular markers of Chloroquine resistance in Douala and Bafoussam 18 Years after the withdrawal of Chloroquine as first line treatment for malaria in Cameroon.**

Application Type:	**Initial**
Sponsor:	**Student**
Review Type:	**Normal**
Date of Approval:	**6th November 2019**
Expiration Date:	**One year**

Principal Investigator's responsibilities:

1. The study must be conducted in strict accordance with the protocol approved by the Board
2. Changes to the protocol or its related consent documents must be approved by the Board before implementation
3. Adverse events or unanticipated problems must be reported promptly to the Board
4. Participants must receive a copy of the consent document, if appropriate
5. The Principal Investigator is responsible for the on-going conduct of the study. The study must be implemented according to national and international guidelines for the ethical conduct of research on humans. He must collaborate with the IRB's monitoring of the study's implementation.
6. Any future correspondence must include the application number, and the PI's name in the subject line.
7. A renewal application or project closure report must be submitted at least one month prior to the expiration date indicated above. These must be done using the FHSIRB's secretariat AND an electronic copy sent to: irbfhs@gmail.com, making sure to reference the application number indicated above. This form is available at http://www.healthresearchweb.rg/en/cameroon/institution2130

Assoc. Prof. Halle Ekane Edie Gregory
Secretary; Institutional Review Board
Faculty of Health Sciences University of Buea
